# Knowledge graph visualization and bibliometric analysis of research on coronary artery lesions in Kawasaki disease

**DOI:** 10.1097/MD.0000000000049678

**Published:** 2026-07-17

**Authors:** Yinglong Huang, Yuzhen Zhang, Jianfei Zhang, Xiaofeng Ma, Ya’nan Zhang

**Affiliations:** a Pediatrics Department, People’s Hospital of Ningxia Hui Autonomous Region, Yinchuan, China; b Department of Surgical Oncology, Shaanxi Provincial People’s Hospital, Xi’an, China.

**Keywords:** bibliometrics, coronary artery lesions, Kawasaki disease, knowledge graph, visual analysis

## Abstract

**Background:**

Coronary artery lesions (CAL) complicating Kawasaki disease (KD) are a central concern affecting the long-term cardiovascular health of affected children. This study aimed to systematically depict the macro landscape, knowledge structure, and evolutionary dynamics of this field through bibliometric and knowledge graph methods, providing strategic guidance for future research.

**Methods:**

Literature related to KD-CAL was retrieved from the Web of Science Core Collection. Quantitative and visual analyses of publication trends, countries/regions, institutions, authors, journals, references, and keywords were conducted using CiteSpace 6.3.R1, VOSviewer 1.6.20, and the Bibliometrix R package.

**Results:**

A total of 2655 publications were included. The annual publication volume showed an increasing trend, with acceleration after 2015. The United States emerged as the country with the highest productivity and influence (citation frequency, centrality), while China ranked first in publication output (909 articles) but had a lower rate of international collaboration (MCP_Ratio = 0.08). The core journal cluster comprised *Circulation*, *Journal of Pediatrics*, and *Pediatric Cardiology*, among others. A close international collaboration network was formed around key figures like Jane Carleton Burns and Adriana H. Tremoulet. Keyword and document co-citation analyses revealed 5 major research clusters: acute-phase management, immunopathological mechanisms, coronary complications, treatment of intravenous immunoglobulin resistance, and long-term follow-up. The research frontier has evolved from “epidemiology” and “diagnosis” to “biomarkers” and “IVIG resistance,” and is now predominantly focused on “biologics” (e.g., infliximab, anakinra) and “artificial intelligence/machine learning.”

**Conclusion:**

The new phase of KD-CAL research may focus on precision medicine. Future efforts may focus on deepening immunological mechanism studies to develop targeted therapies, utilizing artificial intelligence to optimize risk stratification and imaging assessment, and establishing globally collaborative long-term cohorts to clarify cardiovascular outcomes in adulthood. Strengthening basic and clinical translation is essential to advance KD-CAL diagnosis and treatment into a new era of precision and efficiency.

## 1. Introduction

Kawasaki disease (KD) is an acute systemic vasculitis syndrome primarily affecting children under 5 years of age.^[1]^ Its etiology remains unknown but is widely believed to be associated with abnormal immune activation triggered by specific infectious factors in genetically susceptible individuals.^[2]^ Since its first description in 1967, KD has become the leading cause of acquired heart disease in children in many developed countries worldwide.^[3,4]^ The most severe aspect of the disease is its predilection for the coronary arteries, leading to coronary artery lesions (CAL), a spectrum ranging from transient vascular dilation to medium-sized and giant coronary artery aneurysms.^[5–7]^ These lesions are not only central to acute-phase risk assessment but also pose a significant long-term threat to cardiovascular safety. Even with standard treatment (intravenous immunoglobulin [IVIG] combined with aspirin),^[8,9]^ approximately 3% to 5% of children develop coronary artery aneurysms, with some giant aneurysms potentially progressing to stenosis, thrombosis, and ultimately myocardial infarction or sudden cardiac death.^[10]^ This makes KD-CAL a persistent health threat extending from childhood through adolescence and into adulthood.

Over the past 2 decades, alongside rapid advances in immunology, molecular biology, and medical imaging,^[11]^ KD-CAL research has undergone a profound transformation from macroscopic clinical observation to microscopic mechanistic exploration. The research focus has shifted from initial epidemiological descriptions and diagnostic criteria establishment to screening for genetic susceptibility loci, dissecting immune pathogenesis pathways (e.g., interleukin-1, interleukin-6, Ca^2+^ signaling), discovering novel biomarkers, and constructing risk prediction models.^[12–14]^ Concurrently, addressing the clinical challenge of IVIG unresponsiveness, the exploratory application of biologics like infliximab and anakinra marks a strategic shift towards targeted and precise therapy.^[15,16]^ Furthermore, as surviving children reach adulthood, the long-term cardiovascular management of “adult-onset KD” has become an emerging interdisciplinary issue, driving research on long-term follow-up, imaging assessment, and secondary prevention strategies. These multidimensional, cross-scale explorations have led to an explosive growth of scientific literature in this field, forming a vast, diverse, and interconnected complex knowledge system.

However, this dramatic expansion of knowledge also presents new challenges. Researchers, especially newcomers to the field, find it difficult to quickly and systematically grasp its overall developmental trends, core knowledge structures, the lineage of key academic contributions, and future research avenues. Traditional narrative reviews are limited by authors’ subjective perspectives and finite literature coverage, making it challenging to provide an objective, panoramic portrayal of such a vast knowledge domain. In this context, bibliometrics combined with knowledge graph visualization methods offers unique advantages.^[17]^ By quantitatively analyzing (e.g., output, collaboration, citations) and mining relationships (e.g., co-citation) within a large corpus of literature, this approach can objectively reveal the intrinsic laws of disciplinary development, identify core research forces and knowledge bases, dynamically track the evolution of research hotspots, and prospectively detect emerging frontiers.^[18]^

Therefore, this study aims to conduct the first comprehensive and systematic examination of the KD-CAL field using bibliometric and knowledge graph methods. It will quantify the research output trends between 2011 and 2025, map the collaborative networks and influence landscape of countries/regions, institutions, and authors; identify the key scholarly literature (knowledge base) and core research themes (knowledge structure) underpinning the field’s development; visually present the historical evolution of research hotspots; and detect current research frontiers and potential future directions. We hope this study will chart an “academic map” of KD-CAL research, providing an efficient entry guide for new researchers, offering objective data-driven support for scholars within the field to clarify the position of their own work, identify collaboration opportunities, and plan future breakthroughs, thereby collectively advancing the field towards the ultimate goal of eliminating long-term cardiovascular sequelae in children.

## 2. Materials and methods

### 2.1. Data source

The data retrieval for this study was completed on January 15, 2026, with data sourced from the Web of Science Core Collection (SCI-EXPANDED).

### 2.2. Search strategy

The search query was: TS=(“Kawasaki disease” OR “Kawasaki syndrome” OR “Mucocutaneous lymph node syndrome” OR “KD”) AND (“coronary artery” OR “coronary aneurysm*” OR “myocardial ischemia” OR “myocardial infarction”). The time span was from January 1, 2011, to December 31, 2025 (969 records were removed), Document types were limited to Articles and Reviews (331 records were removed), and the language was restricted to English (31 records were removed).

Subsequently, 2 independent reviewers screened the retrieved records to remove duplicates (inter-rater reliability was assessed using Cohen’s kappa coefficient (*k* = 0.94), indicating almost perfect agreement. Discrepancies were resolved through discussion between the 2 reviewers; any remaining disagreements were adjudicated by the corresponding author), irrelevant studies, and low-quality articles (16 records were removed)), resulting in a final set of 2655 publications.

### 2.3. Data analysis and visualization

**Figure 1.**
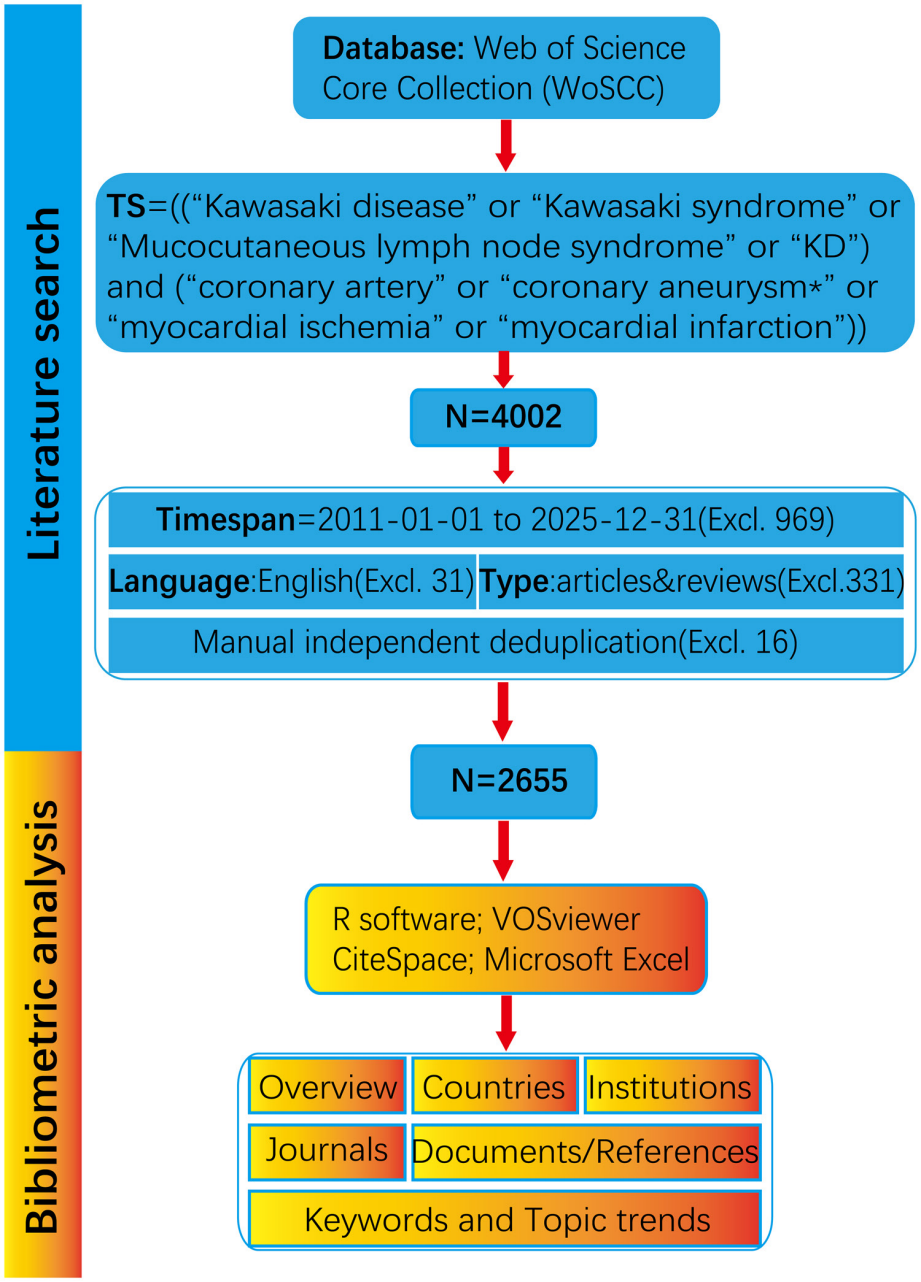
Research flowchart.

CiteSpace 6.3.R1, developed by Chaomei Chen, College of Computing and Informatics, Drexel University,available from http://cluster.cis.drexel.edu/~cchen/citespace/ (parameter settings: time slice = 2 years, node types selected as needed, pruning algorithm = Pathfinder, node retention was configured using a top 50 per slice strategy. Clustering analysis was performed using the log-likelihood ratio algorithm, with a Modularity *Q* threshold >0.5 applied to ensure cluster stability). VOSviewer 1.6.20^[19,20]^ developed by Nees Jan van Eck and Ludo Waltman, Centre for Science and Technology Studies (CWTS), Leiden University, Leiden, the Netherlands, available from https://www.vosviewer.com/ (for co-occurrence mapping, the association strength method was used for normalization, and nodes were weighted by total link strength) and the Bibliometrix R package (an open-source R package developed by Massimo Aria and Corrado Cuccurullo, Department of Business and Management, University of Naples Parthenope, Naples, Italy, available from https://www.bibliometrix.org) were used for data processing, network construction,^[21]^ and visualization. The analysis included: annual publication trends and country/region collaboration networks; journal analysis; author collaboration network analysis; keyword co-occurrence, clustering, and burst detection; and reference citation analysis. The analysis followed a path from macro to micro perspectives, covering the overall profile of total published papers, countries, regions, and institutions. The research process is illustrated in Figure 1.

## 3. Results

### 3.1. Research output trends

**Figure 2.**
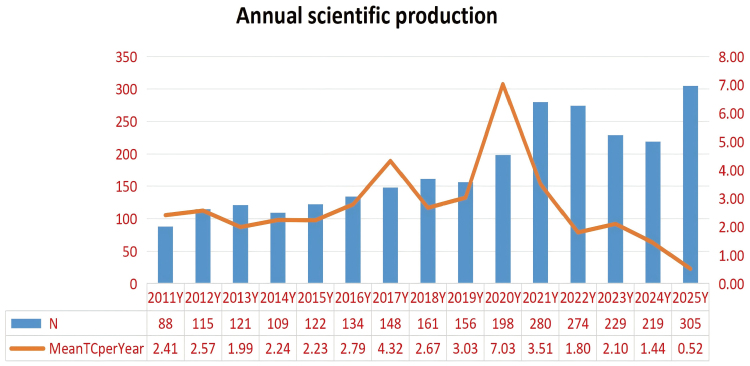
Annual publication output and average citation status of KD-CAL-related research. CAL = coronary artery lesions, KD = Kawasaki disease.

A total of 2655 KD-CAL-related publications, comprising 2275 research articles and 380 reviews, were identified from the Web of Science database. Analysis of the overall publication output in the KD-CAL field revealed a significant exponential growth pattern starting in 2011 (fitting curve *R*^2^ = 0.952). The annual growth rate increased notably after 2015, with a significant rise in publications by 2019, a slight dip in 2023, and another significant increase in 2025. The mean total citations per year showed a slight increase before 2017, followed by a mild decline. A period of rapid growth occurred for 2 years starting in 2019, likely associated with the COVID-19 pandemic. After 2020, the mean total citations per year exhibited a continuous downward trend. This is likely attributable to the diluting effect of the cumulative increase in total publications in the field on citations per paper, coupled with the inherently lower citation counts for recent outputs from 2024 and 2025 due to their short publication history (details shown in Fig. 2).

### 3.2. Country/region collaboration analysis

**Figure 3.**
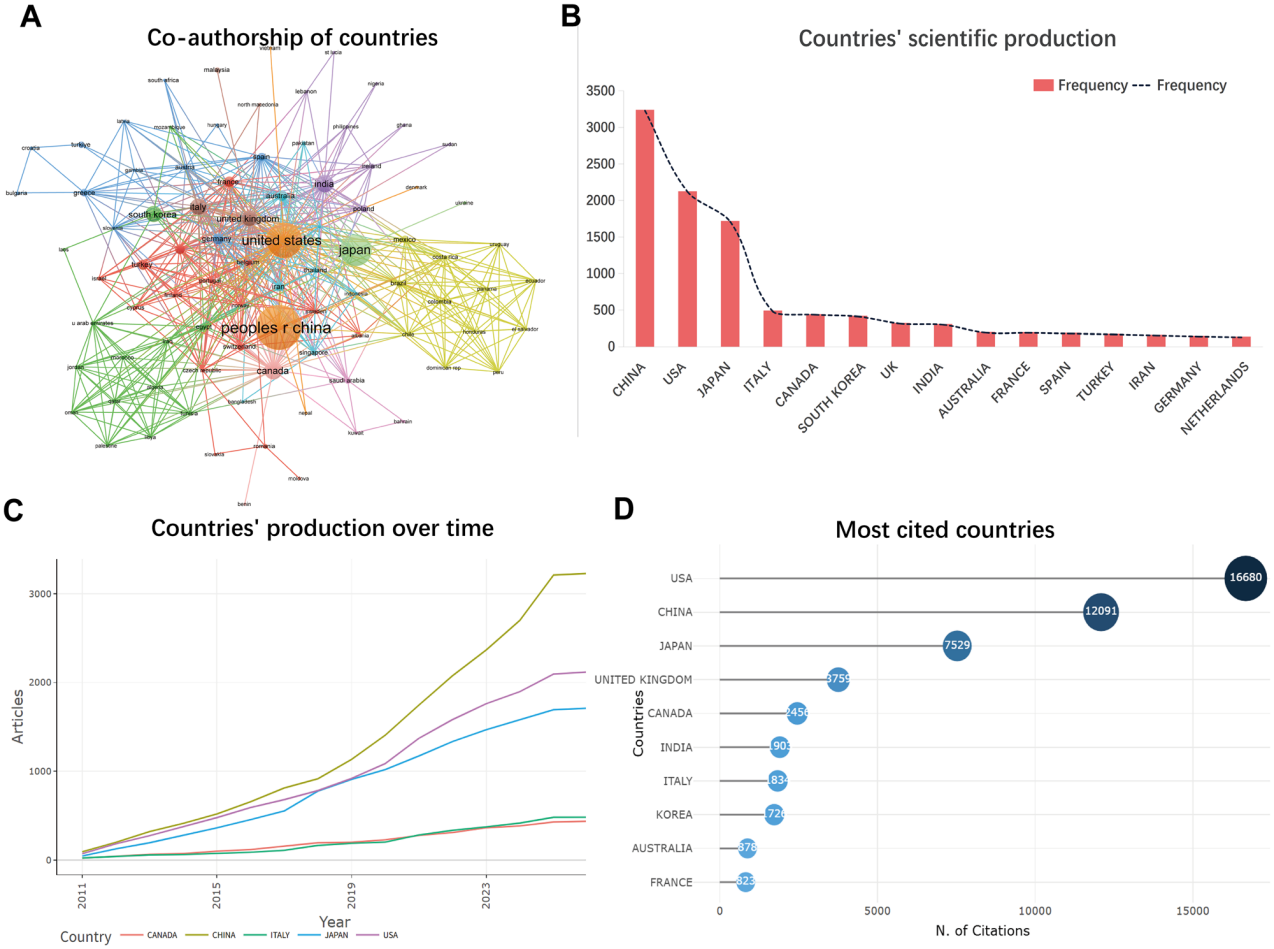
Country/region analysis. (A) Collaboration network of countries/regions involved in the research. (B) Count of publications from each country. (C) Yearly publication patterns of countries/regions. (D) Top 10 most-cited countries.

The publication contributions of various countries involved in this research field were analyzed. The network map illustrating inter-country/region collaborative relationships positioned the United States node at the geometric center, with relatively thick direct connections to multiple major research countries/regions, including China, Japan, Canada, the United Kingdom, Germany, Italy, and the Republic of Korea. This indicates that the United States serves as the central hub of the international collaboration network. In contrast, although the nodes for China and Japan are large (indicating high output), their connections to other countries (especially to each other) appear relatively fewer or thinner (Fig. 3A). This observation aligns with the low “MCP%” data for both countries (China 6.9% and Japan 4.5%) shown in Table 1, suggesting that their research is primarily conducted independently domestically (single-country publication), with room for improvement in the breadth and depth of international collaboration. Countries like Canada and the United Kingdom form a Western collaborative cluster tightly connected to the United States via strong links. The fractional output (where each participating author is counted once) for each country from 2011 to 2025 is shown in Figure 3B, with China having the highest fractional count, potentially related to a larger average number of authors listed per Chinese publication. Figure 3C reveals the historical evolution of research activity by country. The curves for the United States and Japan started earliest and remained at high levels, indicating their status as traditional leaders and sustained contributors. China’s curve started later but showed an extremely steep upward trend around 2015, surpassing or approaching the levels of the United States and Japan in recent years, consistent with its characteristic of “rapid growth as a latecomer,” likely due to increased research investment and rich clinical case resources. Curves for other countries are relatively flat, showing stable but limited growth. Analysis of citation impact by country showed that although China had the highest publication output, its total citation count was significantly lower than that of the United States, which ranked first with 16,680 citations, followed by Japan, the United Kingdom, and others in descending order (Fig. 3D).

**Table 1:** Top 20 corresponding author’s countries.

	Country	Articles	Articles %	SCP	MCP	MCP %
1	China	909	34.2	846	63	6.9
2	United States	420	15.8	317	103	24.5
3	Japan	399	15	381	18	4.5
4	India	121	4.6	108	13	10.7
5	Republic of Korea	111	4.2	105	6	5.4
6	Canada	98	3.7	55	43	43.9
7	Italy	89	3.3	73	16	18
8	United Kingdom	56	2.1	30	26	46.4
9	Turkey	42	1.6	41	1	2.4
10	Iran	36	1.4	33	3	8.3
11	France	32	1.2	21	11	34.4
12	Germany	31	1.2	22	9	29
13	Netherlands	26	1	18	8	30.8
14	Australia	25	0.9	17	8	32
15	Greece	18	0.7	12	6	33.3
16	Poland	18	0.7	17	1	5.6
17	Mexico	17	0.6	13	4	23.5
18	Spain	15	0.6	11	4	26.7
19	Malaysia	11	0.4	10	1	9.1
20	Saudi Arabia	11	0.4	10	1	9.1

MCP = multiple-country publication, SCP = single-country publication.

### 3.3. Distribution of core productive institutions

**Figure 4.**
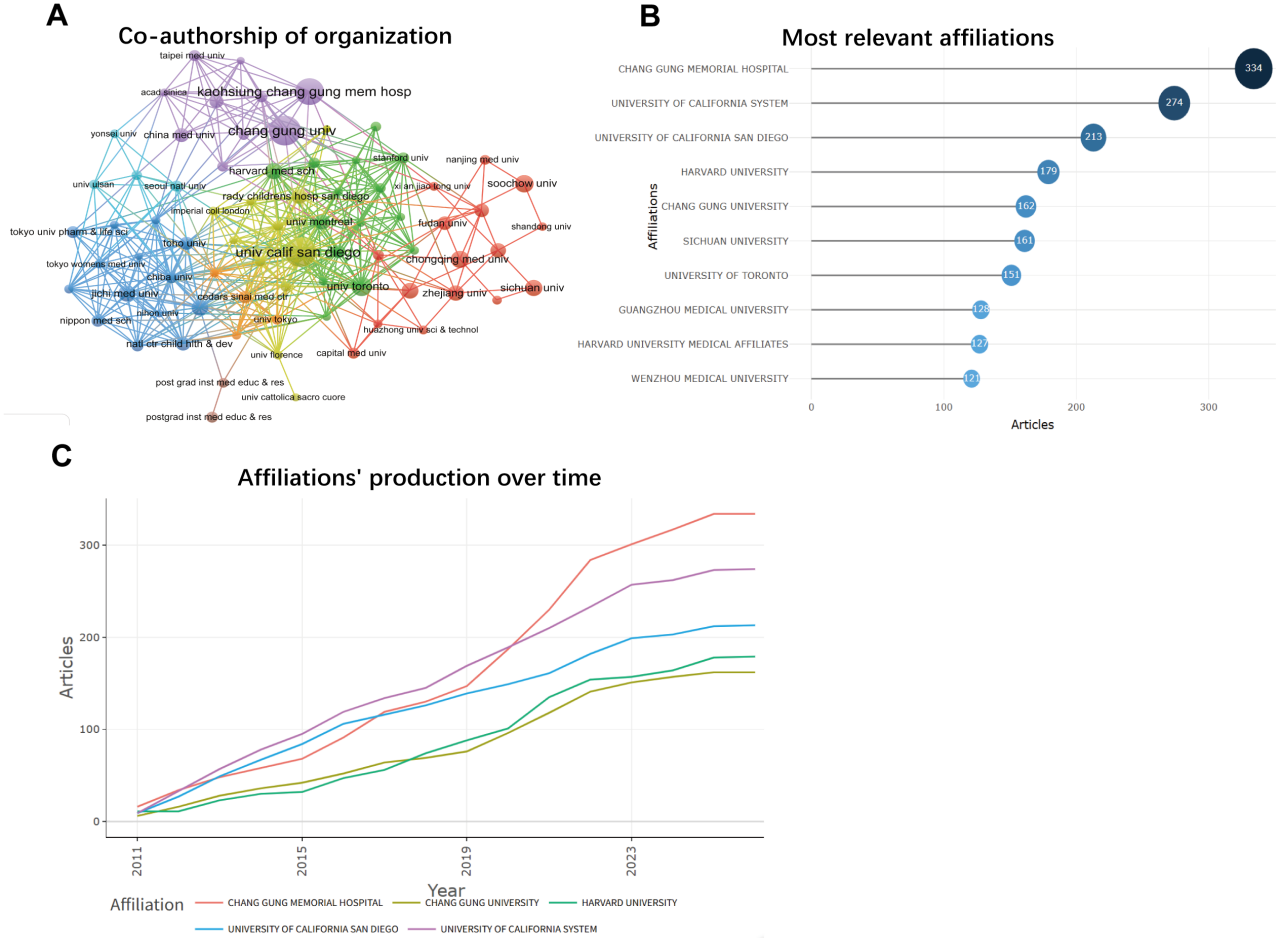
Institutional analysis. (A) Co-authorship network of participating research institutions. (B) Core institutions ranked by publication count. (C) Publication trends of institutions over the past 15 years.

From 2878 global institutions, 77 that published at least 15 papers were selected. Analysis of collaborative relationships between these institutions revealed that world-leading institutions like Harvard Medical School, Chang Gung University, Imperial College London, University of Tokyo, and University of California System, San Diego occupy central or hub positions in the network, playing key roles and highlighting their extensive collaborative ties (Fig. 4A). Analysis of institutions most relevant to the topic indicated that Chang Gung Memorial Hospital led with 274 related articles, demonstrating dominant research activity and influence. University of California System, San Diego (213 articles) and University of California System (179 articles) ranked second and third, respectively, representing the strong research capabilities of top United States public university systems. Harvard University (161 articles), Sichuan University (161 articles), and Chang Gung University (162 articles) formed a dense second tier, each with over 160 articles. Six of the top 10 institutions are from China (including Taiwan), 3 from the United States, and 1 from Canada (Fig. 4B). Analyzing the annual publication output evolution of the top 5 institutions from 2011 to 2025 revealed their trajectories. The output curves for Chang Gung Memorial Hospital and Chang Gung University consistently remained at the highest level with steady growth, confirming their role as long-term, stable, and highly productive core research forces. Harvard University’s curve showed the steepest growth slope, especially after 2019, with its growth rate significantly exceeding all others, indicating a sharp recent increase in research investment and output. The curves for UC San Diego and the UC system showed relatively moderate growth, maintaining medium-level output (Fig. 4C).

### 3.4. Distribution of core journals

**Figure 5.**
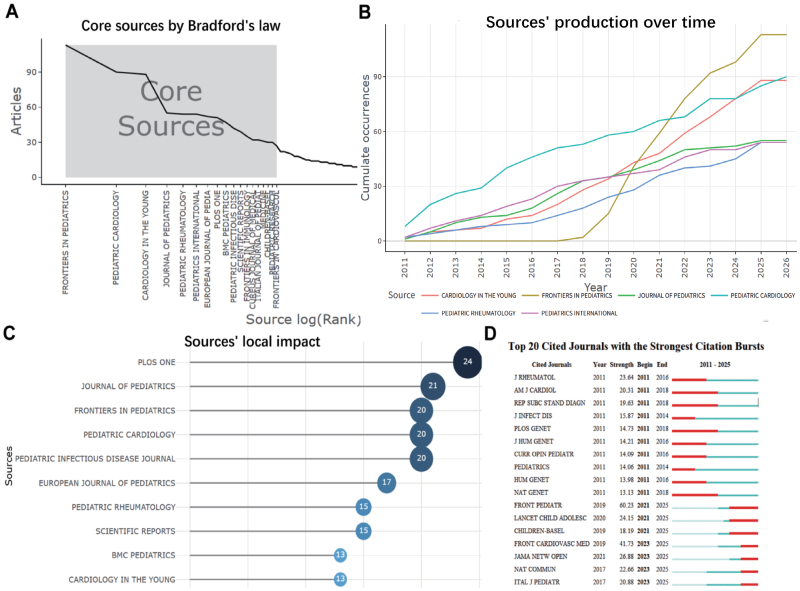
Journal distribution. (A) Distribution of paper counts in core journals. (B) Cumulative trend in the number of related publications from the top 5 core journals (2011–2026). (C) Comparative analysis of journal H-index. (D) Top 20 journals with the strongest citation bursts.

The distribution of core journals, visualized based on Bradford law, highlighted *Frontiers in Pediatrics*, *Pediatric Cardiology*, *Pediatrics*, *Journal of Pediatrics*, and *Cardiology in the Young* as core literature sources. The titles of these core journals clearly indicate the field’s focus on pediatrics, particularly pediatric cardiology, rheumatology, and infectious diseases. The inclusion of interdisciplinary or general journals like *Frontiers in Immunology and Scientific Reports* indicates the field’s strong cross-disciplinary nature (e.g., immunology, basic research; Fig. 5A). Analysis of the cumulative output over time for the top 5 core journals showed a consistent upward trend for all, indicating sustained growth in overall research activity. The curve for specialized journals like *Pediatric Cardiology* showed stable early growth, reflecting its status as a traditional core channel. In contrast, journals like *Frontiers in Pediatrics* exhibited a steeper slope in later years (e.g., post-2020), indicating their key role in accommodating the recent surge in new research (Fig. 5B). Assessing the relative influence of these core journals within the specific dataset revealed *PLOS ONE* as the leader with a “local impact” score of 24, suggesting its articles in this dataset have the strongest average influence (based on metrics like average citations per article). *Journal of Pediatrics* (21), *Frontiers in Pediatrics*, *Pediatric Cardiology*, and *Pediatric Infectious Disease Journal* (all 20) formed a cluster of highly influential core journals (Fig. 5C, details are shown in Table S1, Supplemental Digital Content 1). The “citation burst detection” algorithm identifies journals that were exceptionally highly cited during specific periods, revealing shifts in the research frontier and knowledge base. Traditional high-impact specialty journals like *J Rheumatol*, *Am J Cardiol*, *J Infect Dis*, *Pediatrics*, and *Nat Genet* showed citation bursts relatively early, mostly ending before 2016, constituting the field’s early foundational knowledge. Recent journals like *Front Pediatr*, *Lancet Child Adolesc*, *Front Cardiovasc Med*, *JAMA Netw Open*, and *Nat Commun* mostly exhibited bursts starting after 2020, with many ongoing into 2025. *Front Pediatr* had the strongest burst intensity (60.23), indicating explosive attention. This represents the most active, cutting-edge direction of current research, showing a shift in the knowledge base from traditional classic journals to modern, open-access, rapid-dissemination platforms (Fig. 5D).

### 3.5. Reference analysis

**Figure 6.**
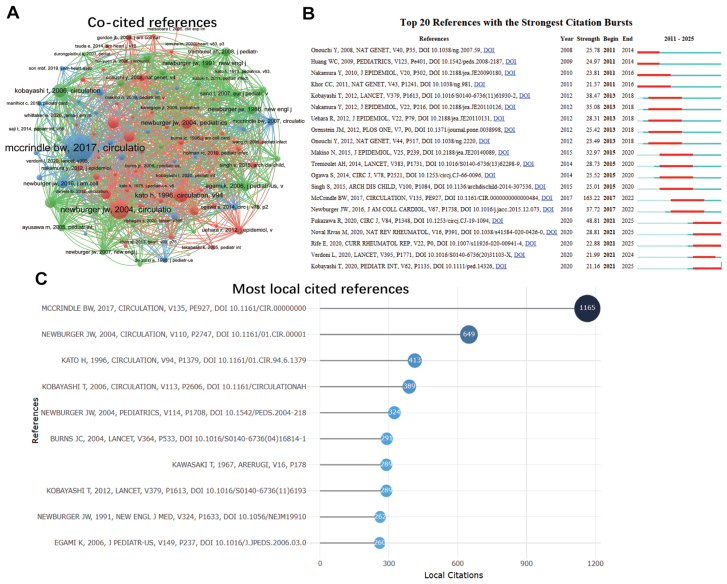
Reference analysis. (A) Co-citation network of references. (B) Top 20 references with the strongest citation bursts. (C) Top 10 most locally cited references.

Analysis of references cited in KD-CAL literature was performed. The co-citation network among references (Fig. 6A) shows that works like McCrindle, 2017, *Circulation*; Newburger, 2004, *Circulation*; Kato, 1996, *Circulation*; and Kobayashi, 2006, *Circulation* occupy relatively central positions with high co-citation frequencies, representing influential research recognized in certain periods. Citation burst detection pinpoints references cited with abnormally high frequency during specific time windows, visually revealing major shifts in the knowledge base, consensus formation, and hotspot migration. For instance, the burst associated with the guideline by McCrindle, 2017, *Circulation* reflects its role as the current standard for clinical practice, defining the global reference benchmark. Similarly, bursts for Kobayashi, 2012, *Lancet*, and Tremoulet, 2014, *Lancet* – clinical trials in *The Lancet*, exploring different IVIG regimens or adding new agents (e.g., infliximab)-mark the field’s critical shift from a “single standard regimen” towards “risk-stratified, personalized therapy” (Fig. 6B). Analysis of the top 10 most-cited references revealed McCrindle, 2017, *Circulation*, as the most cited (1165 citations), its lead underscoring its role as the cornerstone and core reference standard. Newburger, 2004, *Circulation* ranked second (649 citations), representing the previous highly influential AHA guideline that established the standard IVIG treatment regimen, among other contributions (Fig. 6C).

### 3.6. Core authors and collaboration network

**Figure 7.**
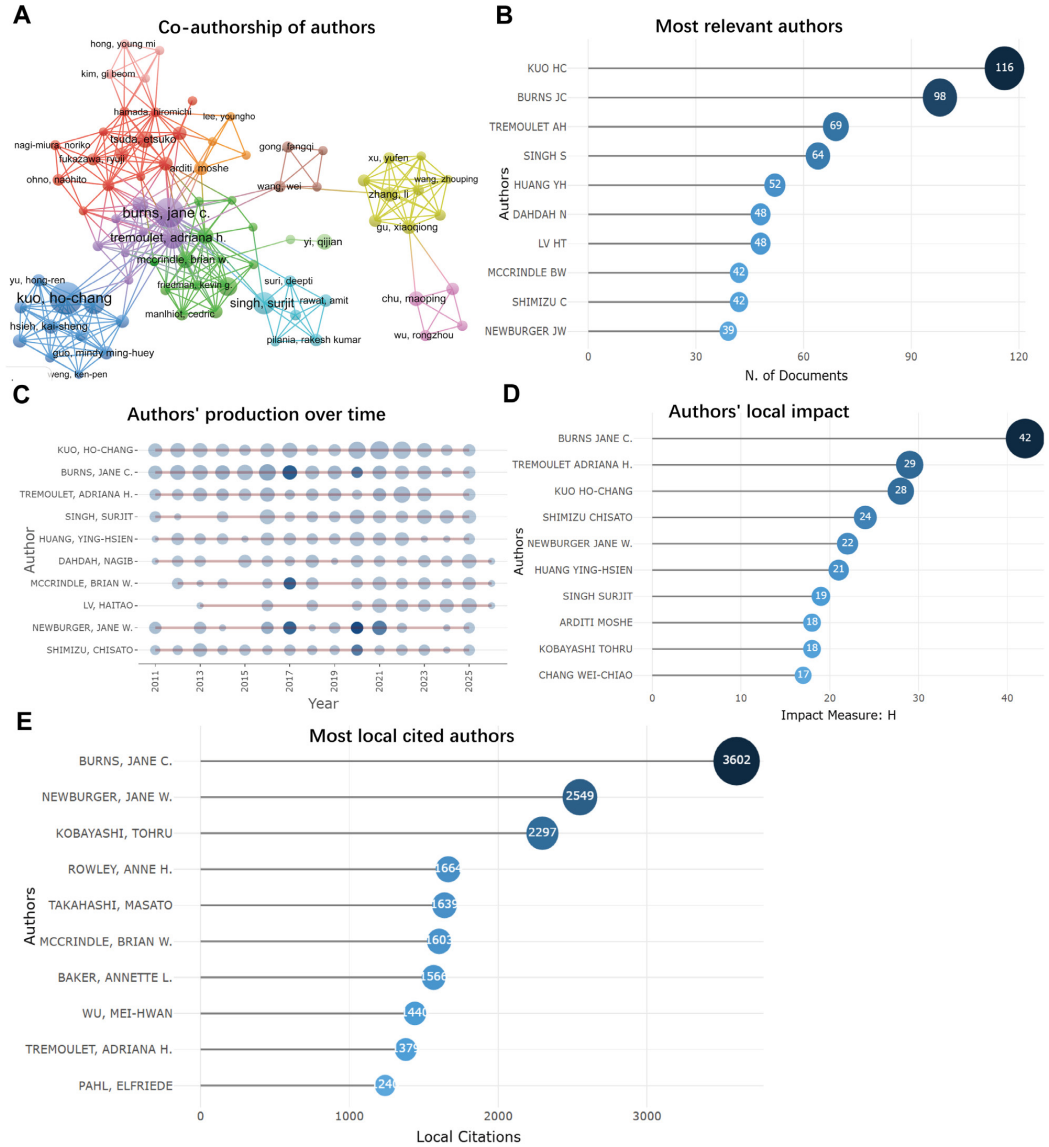
Author-related analysis. (A) Coauthor network. (B) Thematic relevance analysis of authors. (C) Timeline of author publications. (D) Analysis of authors’ local influence based on H-index. (E) Authors’ local citation counts.

An analysis of authors in the KD-CAL field was conducted. The author collaboration network map (Fig. 7A) helps identify core researchers, collaboration patterns, and academic groups; for example, “Jane Carleton Burns” appears as a likely core scholar. Analysis of author productivity showed Ho-Chang Kuo as the most prolific author in the field with 116 publications, far exceeding others. Jane Carleton Burns ranked second with 98 publications, also a core researcher. Authors ranked 3rd to 10th had between 39 and 69 publications (Fig. 7B). Analyzing the yearly publication output of the top 10 authors showed that Ho-Chang Kuo, and Jane Carleton Burns, not only led in total output but also demonstrated remarkable sustained productivity, with their publication bubbles spanning almost the entire timeline, marking them as core drivers of the field’s development (Fig. 7C). Assessing author influence using the H-index within the dataset showed Jane Carleton Burns, with a remarkably high H-index of 42, far ahead of others. This strongly indicates her foundational role, with her work being widely and persistently cited. Adriana Helena Tremoulet, ranked second in influence, followed by Ho-Chang Kuo. Notably, while Ho-Chang Kuo ranked first in productivity, he ranked third in influence, suggesting that high productivity does not always equate to the strongest influence. Ho-Chang Kuo, may be a highly productive author, whereas Jane Carleton Burns’s work holds more “classic” and “foundational” value (Fig. 7D, details are shown in Table S2, Supplemental Digital Content 2). Local citations (citations from within the dataset) are an important metric of an author’s impact. In this study, Jane Carleton Burns’s work was cited 3602 times locally, making her the most cited and central knowledge source. Ho-Chang Kuo, and Adriana Helena Tremoulet, despite their high influence indices, did not show as dominant a lead in local citation counts (Fig. 7E).

### 3.7. Keyword analysis

**Figure 8.**
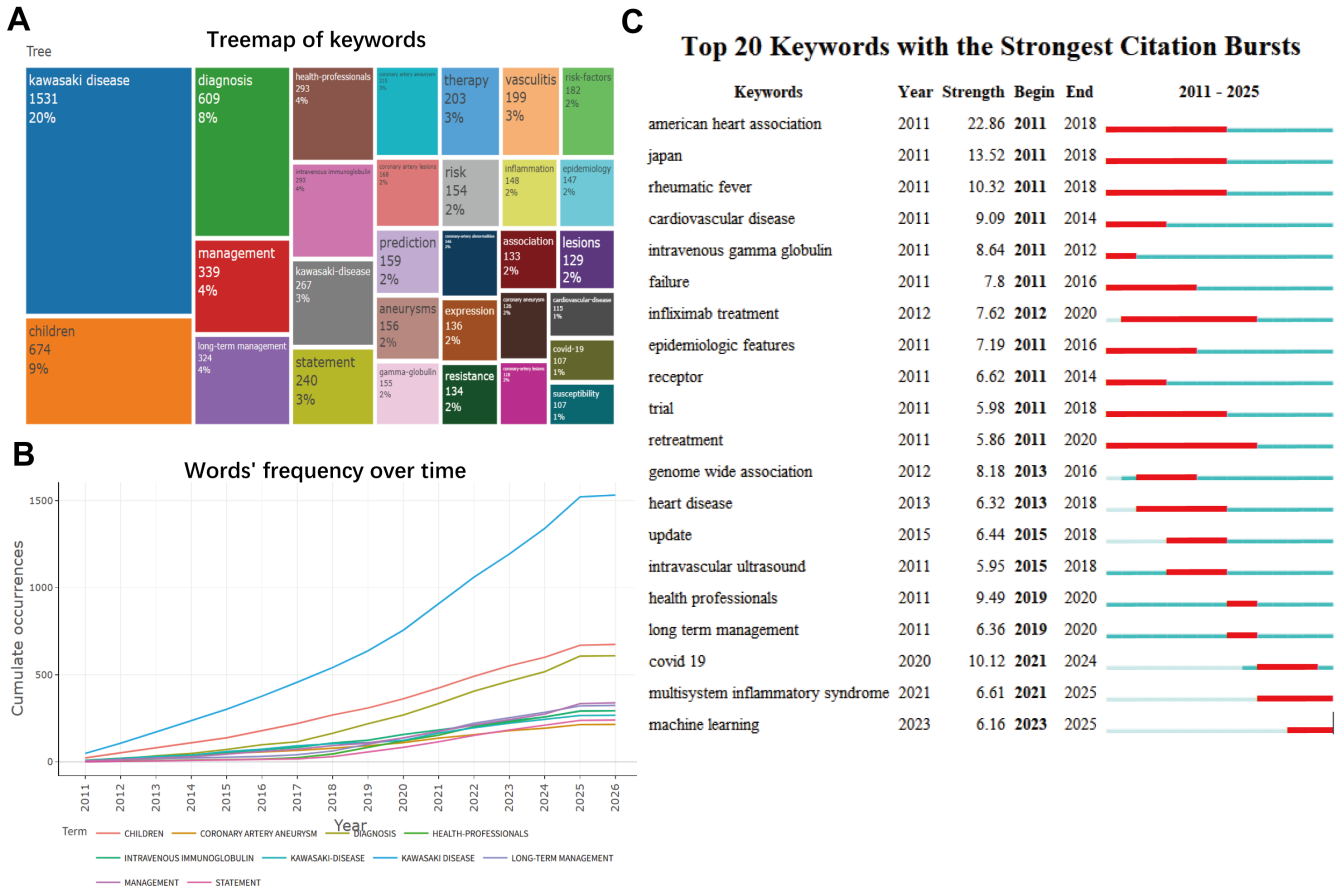
Keyword analysis and research trends. (A) Tree map of the most frequently occurring keywords. (B) Trend of keyword frequency over time. (C) Top 20 keywords with the strongest citation bursts.

To explore research hotspots and future directions, an in-depth analysis of keywords and their trends was conducted. The word cloud (Fig. 8A) visually displays the most frequent keywords, with core topic terms like “Kawasaki disease,” “children,” and “Kawasaki-disease” appearing most frequently (>30%). High-frequency terms like “diagnosis,” “intravenous immunoglobulin,” “gamma-globulin,” “management,” and “long-term management” indicate that diagnosis, differentials, treatment, and disease management remain persistent research priorities. Keywords like “angioplasty” and “cerebrovascular disease” point to interventions and prognosis of critical complications. Terms like “expression” and “susceptibility” reflect exploration into basic mechanisms. The appearance of “COVID-19” is highly time-specific, reflecting recent frontier research on the association between KD, severe acute respiratory syndrome coronavirus 2 infection, and multisystem inflammatory syndrome in children (MIS-C). The temporal evolution of keyword frequencies (Fig. 8B) visually demonstrates the dynamic shifts in research focus from 2011 to 2025. “Kawasaki disease” as the core term shows exponential cumulative growth, nearing 1500 occurrences by 2025, far outpacing others, indicating rapid expansion of the field. Terms like “children,” “diagnosis,” “intravenous immunoglobulin,” “coronary artery aneurysm,” and “long-term management” show relatively parallel, gradual growth, representing stable, persistent sub-directions. Early keyword bursts (before 2012) included “American Heart Association” (strongest: 22.86), “Japan,” “rheumatic fever,” and “trial.” This phase focused on establishing an authoritative framework, referencing AHA guidelines, Japanese (high-incidence region) research, and differential diagnosis with rheumatic fever, reflecting efforts to standardize diagnosis, treatment consensus, and epidemiological foundations – an era of “setting standards.” Post-2012 bursts included terms like “infliximab treatment,” “retreatment,” “genome-wide association,” and “receptor.” With consensus established, research deepened towards optimizing/extending treatments and exploring pathogenesis. The burst of “intravascular ultrasound” represents a focus on refined assessment techniques for coronary complications (Fig. 8C).

### 3.8. Research trends

**Figure 9.**
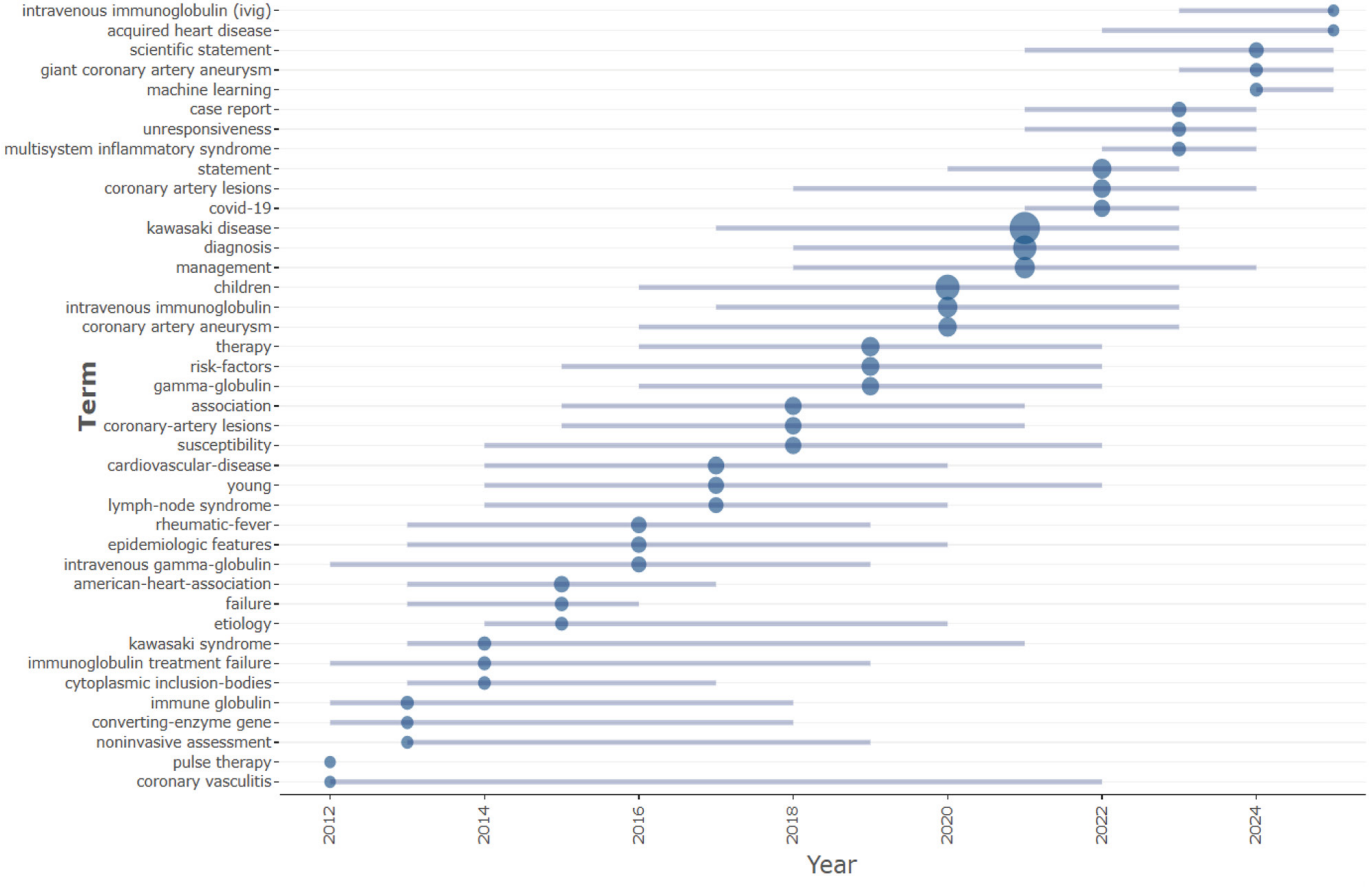
Analysis of thematic trend evolution.

Further analysis of research trends in the field was performed. Around 2012 to 2015, bursts occurred for terms like “American Heart Association,” “rheumatic fever,” “gamma-globulin,” “Kawasaki syndrome,” “lymph node syndrome,” “immunoglobulin treatment failure,” “pulse therapy,” and “coronary vasculitis.” This terminology reflects a phase of framework establishment, definition clarification, and standard formation, referencing guidelines, differential diagnosis, using traditional nomenclature, and exploring initial treatments. Around 2014 to 2018, bursts were seen for “unresponsiveness,” “coronary artery lesions,” “risk factors,” “noninvasive assessment,” and “intravenous immunoglobulin.” This signifies a move towards refining clinical practice post-consensus, shifting focus from “whether to treat” to “how to treat better” (addressing IVIG unresponsiveness, identifying risk factors) and “how to assess more accurately” (noninvasive assessment of CALs)-marking a transition to individualized patient management. Around 2018 to 2020, bursts appeared for “long-term management,” “cardiovascular disease,” “susceptibility,” “acquired heart disease,” “failure,” and “etiology.” This indicates an extension of the research horizon from the acute phase to lifelong management, emphasizing long-term cardiovascular outcomes and a deepening exploration of disease origins. From around 2020 to the present, the latest burst keywords are “COVID-19,” “MIS-C (Multisystem Inflammatory Syndrome in Children),” “children,” “infliximab,” “anakinra,” and “machine learning,” which may defines the current frontier. The COVID-19 pandemic placed KD research in a new global public health context, spurring comparative studies with MIS-C. The “post-COVID” era is focused on deeply comparing the immunopathology, clinical features, and treatment of classic KD versus MIS-C, representing a prolific and urgent frontier. Embracing the “AI revolution,” using machine/deep learning to build risk prediction models, assist imaging diagnosis, and achieve patient subtyping might be a decisive direction propelling the field towards precision medicine (Fig. 9).

## 4. Discussion

This study is the first to employ a combined bibliometric and knowledge graph approach to provide a panoramic, dynamic quantitative scan and visual representation of the research field on KD-CAL. Through systematic analysis of 2655 core publications over the past decade and a half (2011–2025), we have not only quantified the macro developmental trends of the field but also deeply revealed its intrinsic knowledge structure, driving mechanisms, and evolutionary logic. This discussion aims to move beyond simple data description, integrating and interpreting the findings to extract their academic significance, thereby providing scholars with a “strategic map” possessing both historical depth and future orientation.

This study reveals that KD-CAL research has formed a highly active yet unevenly developed global academic ecosystem. The United States, Japan, and China constitute the “triad” of the field, but with distinct roles and contribution patterns. The United States, with the highest total citation count, the strongest betweenness centrality, and a broad collaboration network centered on scholars like Jane Carleton Burns and Adriana H. Tremoulet, firmly occupies the position of the global hub for academic influence and knowledge integration. This leadership is not incidental but stems from its long-standing accumulation in basic immunology and clinical research methodology, coupled with the authority of leading international guideline development (e.g., AHA Scientific Statements^[22]^). In contrast, Japan, as a high-incidence country and an early research leader, has made sustained and stable contributions, though its centrality in recent international collaboration networks appears relatively weaker, possibly reflecting a greater focus on deep analysis of domestic clinical cohorts.

China’s performance is particularly striking and thought-provoking. It ranks first globally in absolute publication output and holds 6 positions among the top 10 institutions by output, with the Chang Gung healthcare system demonstrating remarkable productivity. This undoubtedly benefits from China’s vast clinical case resources, continuously increasing research investment, and rapidly growing research capacity. However, in stark contrast, China’s international collaboration rate (MCP_Ratio = 0.08) is significantly lower, and its average citation impact lags behind that of the United States. This reveals a key issue: “high output” does not directly equate to “high impact.” While China’s KD-CAL research has achieved a leap in “volume,” challenges remain in climbing the ladder of “quality” and “international discourse power.” This may relate to research innovativeness, the proportion published in high-impact journals, and the capacity to initiate or deeply participate in multinational, multicenter studies. Moving forward, Chinese scholars urgently need to shift from “scale-driven” to “quality-and-innovation-driven” research. By more proactively integrating into the United States- and Europe-centric global collaboration networks – not merely as data contributors but as research designers and thought leaders – they can transform the advantage of case numbers into the academic advantage of defining international frontiers.

Author collaboration network analysis clearly indicates that the KD-CAL field is not a loose collection of individual studies but a tightly collaborative system revolving around a few key scholars. This “hub-and-spoke” structure, with the Burns and Tremoulet teams at the core, connects numerous United States research groups globally. This model facilitates efficient resource integration, standardization of research protocols (e.g., case definitions, endpoint adjudication), and rapid advancement of large clinical trials (e.g., infliximab studies) and consensus United States development. However, it may also create a degree of “path dependence,” where the research interests of these core scholars significantly shape the mainstream direction of the field.

Multidimensional assessment of author influence (productivity, H-index, local citations) provides a nuanced perspective on different scholarly roles. The case of Jane Carleton Burns is exemplary. Her exceptionally high H-index and overwhelmingly dominant local citation count, consistent with her identity as a core developer of early guidelines identified in keyword burst analysis, underscore her irreplaceable role as the “knowledge cornerstone” of the field. Her work defines standards and is thus cited by almost all subsequent research.^[23]^ In contrast, scholars like Ho-Chang Kuo, through high output and extensive collaboration, serve as the “engine” driving the field’s expansion and following hot topics.^[24,25]^ The incomplete overlap between highly productive and highly influential authors suggests that evaluating scholarly contribution requires moving beyond simplistic quantitative metrics, simultaneously respecting the depth of foundational knowledge creation and the breadth of contribution through expansion.

Reference co-citation and burst analyses further reinforce this point, indicating that core documents like the 2017 AHA Scientific Statement form the immutable “knowledge foundation” of current research,^[26]^ while landmark clinical trials published in *The Lancet* signify key paradigm shifts in treatment strategies.^[27]^

A core contribution of this study is the systematic reconstruction of the dynamic evolution of KD-CAL research themes through keyword co-occurrence, clustering, and timeline analysis. This evolution is not linear but presents a clear, logically staged developmental trajectory, reflecting 4 significant paradigm shifts in the field’s cognition. The first stage focused on standardization and consensus-building. Keyword bursts during this period (e.g., *American Heart Association, rheumatic fever*) indicate the research community’s primary task was to “understand the disease” and “establish rules.” Differentiating from conditions like rheumatic fever and referencing authoritative guidelines aimed to unify diagnostic language and therapeutic baselines, creating a comparable platform for all subsequent research. This stage laid the foundation for the field’s maturity. The second stage focused on refinement and personalization. After standards were established, the focus naturally moved to “doing it better.” Bursts of keywords like *unresponsiveness*, *risk factors*, and *noninvasive assessment mark* a profound shift from a “one-size-fits-all” treatment approach towards a “risk-stratified, precision intervention” model. The focus on IVIG unresponsiveness created new therapeutic needs, while advances in risk assessment and noninvasive imaging provided tools for individualized management. The third stage focused on deepening and extending to lifelong care. The research horizon expanded in both time and scientific depth. Temporally, bursts of *long-term management and cardiovascular diseases* signify the field’s systematic address of cardiovascular risks as children reach adulthood, bridging pediatric and adult cardiology. Scientifically, bursts of *etiology and susceptibility* indicate a concurrent acceleration in exploring the disease’s origins – genetic and immune mechanisms. The fourth stage focuses on integration and reconceptualization. The COVID-19 pandemic became a significant influential event.^[28]^ The explosive emergence of COVID-19 and MIS-C is not merely an added research association but recontextualizes KD. It prompts researchers to reexamine KD’s immunological essence within a broader spectrum of “MIS-C.” This comparative research, in turn, deepens the understanding of KD’s own mechanisms. Simultaneously, the burst of *machine learning* holds paradigmatic revolutionary potential.^[29,30]^ It signals a shift in the field’s methodology from traditional hypothesis-driven epidemiology and statistics towards data-driven, exploratory artificial intelligence analytics capable of handling high-dimensional complex relationships. This offers unprecedented opportunities to decipher disease heterogeneity and achieve ultra-early prediction and automated imaging analysis.

Based on the above analysis, the KD-CAL field might be charting a course towards “precision medicine.” Current frontiers are highly concentrated in 2 directions: first, comparative immunology of KD/MIS-C, representing a golden window to understand the essence of the disease; and second, artificial intelligence-assisted clinical decision-making, a key technological tool for realizing precision medicine. However, reaching this goal faces multiple challenges. Global health disparities and data gaps cannot be ignored. The geographical imbalances in research output and influence revealed in this study reflect underlying disparities in healthcare resources and research capacity. Establishing truly inclusive, equitable global collaborative networks, sharing data and biospecimens, and particularly focusing on the disease burden in low- and middle-income countries, are crucial for obtaining a complete picture of the disease and developing universally applicable solutions.

As a typical quantitative analytical method, bibliometrics is widely used in disciplinary development analysis and research performance evaluation. However, the method itself has inherent limitations that must be fully acknowledged. First, its reliance on surface-level metrics (e.g., term co-occurrence, citation counts) makes it difficult to access the deeper motivations and logical connections behind knowledge evolution. Furthermore, necessary simplifications during model construction may lead to deviations between analytical results and complex academic realities. Second, the current analysis relies exclusively on the Web of Science database. The exclusion of other comprehensive databases – such as PubMed/MEDLINE, Scopus, and Embase – inevitably limits the international representativeness of our findings. Furthermore, the restriction to English-language publications overlooks research published in native languages within high-incidence regions like Japan and the Republic of Korea. Crucially, this may omit seminal works of significant impact in this field. Consequently, this introduces a systemic disciplinary bias that affects the sample’s generalizability. We acknowledge this as one of the most critical limitations of the present study. Future research should employ a more exhaustive retrieval strategy, incorporating regional databases (e.g., J-Medical, Korean databases) to mitigate bias and enhance the international robustness of the conclusions. Third, the prevailing evaluation system still overly relies on traditional citation metrics, failing to adequately incorporate emerging forms of scientific output like datasets, code, and experimental protocols, or alternative metrics like online attention and societal impact, limiting its applicability in assessing interdisciplinary and open science practices. Fourth, the search timeframe for this analysis (2011–2025) excluded foundational studies published prior to 2011 that established the diagnostic criteria and treatment protocols for KD. This omission may have partially influenced the construction of the overall knowledge framework. Finally, due to relatively slow updates of technical tools and a lack of theoretical frameworks tailored to the characteristics of social sciences and humanities, the applicability of bibliometrics in these fields is often questioned, potentially even encouraging a simplistic “quantity-over-quality” evaluation tendency. To enhance the rigor and explanatory power of such analyses, future efforts should emphasize the integration of qualitative and quantitative research, develop techniques for fusing multisource, heterogeneous scientific data, construct evaluation indicators suited to different disciplinary characteristics, and improve the depth, reliability, and validity of bibliometric studies through methodological innovation and technical standardization.

## 5. Conclusion

This study, utilizing bibliometric and knowledge graph methods, systematically reveals the evolutionary trajectory, knowledge structure, and frontier dynamics of research on KD-CAL. The findings indicate that the field has entered a phase of rapid development since 2015, forming a globally active, multidisciplinary landscape with the United States as the core of academic influence and collaboration, and China as the leading producer of publications. And one of current research trends appear to lean toward immunologic comparisons driven by the COVID-19 pandemic – specifically regarding the link between severe acute respiratory syndrome coronavirus 2 and MIS-C – as well as exploratory efforts to transition toward artificial intelligence-based precision diagnostics and therapeutics. Comparative immunological studies of KD and MIS-C, alongside machine learning-driven data model construction, collectively define the emerging trends of the field.

## Acknowledgments

All claims expressed in this article are solely those of the authors and do not necessarily represent those of their affiliated organizations, or those of the publisher, the editors, and the reviewers. Any product that may be evaluated in this article, or claim that may be made by its manufacturer, is not guaranteed or endorsed by the publisher.

## Author contributions

**Investigation:** Yinglong Huang.

**Methodology:** Yinglong Huang, Yuzhen Zhang, Jianfei Zhang, Xiaofeng Ma.

**Resources:** Yinglong Huang, Yuzhen Zhang, Xiaofeng Ma, Ya’nan Zhang.

**Software:** Yinglong Huang, Yuzhen Zhang, Ya’nan Zhang.

**Supervision:** Yinglong Huang, Xiaofeng Ma.

**Validation:** Yinglong Huang, Xiaofeng Ma.

**Visualization:** Yinglong Huang, Yuzhen Zhang, Xiaofeng Ma.

**Formal analysis:** Yuzhen Zhang, Xiaofeng Ma, Ya’nan Zhang.

**Funding acquisition:** Jianfei Zhang.

**Data curation:** Ya’nan Zhang.

**Project administration:** Ya’nan Zhang.

**Writing – original draft:** Yinglong Huang, Yuzhen Zhang.

**Writing – review & editing:** Yinglong Huang, Yuzhen Zhang, Jianfei Zhang, Xiaofeng Ma, Ya’nan Zhang.




