# Supplementary material for: Knowledge graph visualization and bibliometric analysis of research on coronary artery lesions in Kawasaki disease
Source: Medicine (Baltimore). 2026 Jul 17;105(29):e49678. doi: 10.1097/MD.0000000000049678 (PMC13384709; doi:10.1097/MD.0000000000049678)
Supplement: Supplementary file 1 [file medi-105-e49678-s001.docx]

**Table S1.** Local impact of top 20 sources

| Source | H_index | G_index | M_index |
| --- | --- | --- | --- |
| PLOS ONE | 24 | 40 | 1.5 |
| JOURNAL OF PEDIATRICS | 21 | 40 | 1.313 |
| FRONTIERS IN PEDIATRICS | 20 | 32 | 2.222 |
| PEDIATRIC CARDIOLOGY | 20 | 31 | 1.25 |
| PEDIATRIC INFECTIOUS DISEASE JOURNAL | 20 | 32 | 1.25 |
| EUROPEAN JOURNAL OF PEDIATRICS | 17 | 34 | 1.063 |
| PEDIATRIC RHEUMATOLOGY | 15 | 25 | 0.938 |
| SCIENTIFIC REPORTS | 15 | 25 | 1.25 |
| BMC PEDIATRICS | 13 | 20 | 1 |
| CARDIOLOGY IN THE YOUNG | 13 | 19 | 0.813 |
| FRONTIERS IN IMMUNOLOGY | 13 | 31 | 1 |
| INTERNATIONAL JOURNAL OF RHEUMATIC DISEASES | 13 | 21 | 1.182 |
| KOREAN CIRCULATION JOURNAL | 13 | 18 | 0.813 |
| PEDIATRICS | 13 | 14 | 0.867 |
| ARCHIVES OF DISEASE IN CHILDHOOD | 12 | 16 | 0.75 |
| JOURNAL OF THE AMERICAN HEART ASSOCIATION | 12 | 18 | 1 |
| PEDIATRIC RESEARCH | 12 | 19 | 0.8 |
| PEDIATRICS INTERNATIONAL | 12 | 24 | 0.75 |
| RHEUMATOLOGY INTERNATIONAL | 12 | 19 | 0.75 |
| CLINICAL AND EXPERIMENTAL IMMUNOLOGY | 11 | 14 | 0.688 |
