# Supplementary material for: Knowledge graph visualization and bibliometric analysis of research on coronary artery lesions in Kawasaki disease
Source: Medicine (Baltimore). 2026 Jul 17;105(29):e49678. doi: 10.1097/MD.0000000000049678 (PMC13384709; doi:10.1097/MD.0000000000049678)
Supplement: Supplementary file 2 [file medi-105-e49678-s002.docx]

**Table S2.** Local impact of top 20 authors

|  | Author | H_index | G_index | M_index |
| --- | --- | --- | --- | --- |
| 1 | BURNS JANE C. | 42 | 92 | 2.625 |
| 2 | TREMOULET ADRIANA H. | 29 | 51 | 1.813 |
| 3 | KUO HO-CHANG | 28 | 39 | 1.75 |
| 4 | SHIMIZU CHISATO | 24 | 39 | 1.5 |
| 5 | NEWBURGER JANE W. | 22 | 39 | 1.375 |
| 6 | HUANG YING-HSIEN | 21 | 28 | 1.313 |
| 7 | SINGH SURJIT | 19 | 30 | 1.188 |
| 8 | ARDITI MOSHE | 18 | 30 | 1.2 |
| 9 | KOBAYASHI TOHRU | 18 | 29 | 1.125 |
| 10 | CHANG WEI-CHIAO | 17 | 24 | 1.063 |
| 11 | DAHDAH NAGIB | 17 | 28 | 1.063 |
| 12 | MCCRINDLE BRIAN W. | 17 | 40 | 1.133 |
| 13 | YANG KUENDER D. | 17 | 27 | 1.063 |
| 14 | KUIJPERS TACO W. | 16 | 22 | 1 |
| 15 | NAKAMURA YOSIKAZU | 16 | 27 | 1.067 |
| 16 | YU HONG-REN | 16 | 24 | 1 |
| 17 | LEVIN MICHAEL | 15 | 18 | 0.938 |
| 18 | MANLHIOT CEDRIC | 15 | 23 | 1 |
| 19 | DIONNE AUDREY | 14 | 29 | 1.167 |
| 20 | HSIEH KAI-SHENG | 14 | 22 | 0.875 |
